# OSgbm: An Online Consensus Survival Analysis Web Server for Glioblastoma

**DOI:** 10.3389/fgene.2019.01378

**Published:** 2020-02-21

**Authors:** Huan Dong, Qiang Wang, Ning Li, Jiajia Lv, Linna Ge, Mengsi Yang, Guosen Zhang, Yang An, Fengling Wang, Longxiang Xie, Yongqiang Li, Wan Zhu, Haiyu Zhang, Minghang Zhang, Xiangqian Guo

**Affiliations:** ^1^ Department of Predictive Medicine, Institute of Biomedical Informatics, Cell Signal Transduction Laboratory, Bioinformatics Center, Henan Provincial Engineering Center for Tumor Molecular Medicine, School of Software, School of Basic Medical Sciences, Henan University, Kaifeng, China; ^2^ Department of Anesthesia, Stanford University School of Medicine, Stanford, CA, United States; ^3^ Department of Pathology, Stanford University School of Medicine, Stanford, CA, United States; ^4^ Nanjing Jiliang Biotechnology Co., Ltd., Nanjing, China

**Keywords:** glioblastoma, survival analysis, prognostic biomarker, OSgbm, transcriptome profiles, clinical information

## Abstract

Glioblastoma (GBM) is the most common malignant tumor of the central nervous system. GBM causes poor clinical outcome and high mortality rate, mainly due to the lack of effective targeted therapy and prognostic biomarkers. Here, we developed a user-friendly Online Survival analysis web server for GlioBlastoMa, abbreviated OSgbm, to assess the prognostic value of candidate genes. Currently, OSgbm contains 684 samples with transcriptome profiles and clinical information from The Cancer Genome Atlas (TCGA), Gene Expression Omnibus (GEO) and Chinese Glioma Genome Atlas (CGGA). The survival analysis results can be graphically presented by Kaplan-Meier (KM) plot with Hazard ratio (HR) and log-rank *p* value. As demonstration, the prognostic value of 51 previously reported survival associated biomarkers, such as *PROM1* (HR = 2.4120, *p* = 0.0071) and *CXCR4* (HR = 1.5578, *p < *0.001), were confirmed in OSgbm. In summary, OSgbm allows users to evaluate and develop prognostic biomarkers of GBM. The web server of OSgbm is available at http://bioinfo.henu.edu.cn/GBM/GBMList.jsp.

## Introduction

Glioblastoma (GBM) is the most common malignant tumor of the central nervous system (CNS) and causes a high mortality rate (Nikiforova and Hamilton, 2011; Stoyanov et al., 2018). Although many new therapies have improved the clinical outcome and more clinical trials have demonstrated the high efficacy in treating GBM, the survival rate of GBM patients is still low. GBM is a complex disease to tackle with a median survival period of approximately 14 months, and a 5-year survival rate of 5% (Stupp et al., 2005; Johnson and O'Neill, 2012; Polivka et al., 2017). Prognostic biomarkers have been showing great roles in cancer patient management and may guide targeted therapies. Therefore, it is greatly needed to investigate prognostic biomarkers in GBM.

Previous studies have reported some prognostic biomarkers in GBM, such as gene mutation of gene *IDH* and *PTEN*, and expression variation of gene *CD133* (Yang et al., 2016; Cai and Sughrue, 2017; Nguyen et al., 2018). However, these biomarkers have not been translated to clinical applications due to the lack of independent validation. In addition, due to the molecular heterogeneity among GBMs and limited patient samples (Nathanson et al., 2014; Aldape et al., 2015; Brown et al., 2017), the prognostic behavior of a certain biomarker may be inconsistent or even contradictory between different reports. In other words, cross population validation in a larger patient cohort is critical for evaluating the prognostic biomarker.

In current work, we collected the gene expression profiles and clinical information of 684 GBM patients from seven independent cohorts obtained from TCGA, GEO and CGGA. We developed a user-friendly web server, OSgbm, to analyze the prognostic value of genes of interests. With this web server, it would facilitate researchers and clinicians to screen, develop and validate new prognostic biomarkers in GBM.

## Methods

### Datasets Collection

GBM datasets are from three major data sources. First, level-3 gene expression profiling data (HiSeqV2) and clinical information of GBM samples were downloaded from TCGA on April 2018 (https://portal.gdc.cancer.gov/). Second, four cohorts (≥30 cases) with available gene expression profiles and clinical survival information were collected from GEO database (http://www.ncbi.nlm.nih.gov/geo/). Third, two GBM cohorts were gathered from CGGA (http://www.cgga.org.cn/). After an initial filtration and quality check (with available gene expression profiling data and clinical survival information), 153 samples from TCGA, 276 samples from GEO, and 255 samples from CGGA were included for the following database and web server construction. The histology of recurrent GBM (rGBM) were included in GSE7696 (10 samples), GSE42669 (11 samples), CGGAarray (9 samples) and CGGAseq (22 samples) datasets. Two CGGA datasets also included 20 samples of secondary GBM (sGBM).

### System Implementation and Server Set-Up

OSgbm is a web-based tool which uses J2EE (Java 2 Platform Enterprise Edition) architecture as we previously described (Wang et al., 2019; Wang et al., 2019; Xie et al., 2019a; Zhang et al., 2019). The gene expression and clinical data were integrated in the background database, which was handled by a MySQL server. Dynamic web interfaces were written in HTML 5.0 and hosted by Tomcat on Windows Server. Using OSgbm requires a HTML 5.0-compliant browser with JavaScript enabled, but does not require any particular visual plug-in tool. Since the web server was designed for users with no specialized bioinformatics skills, we propose ‘out-of-the-box’ data. The input of OSgbm web server is official gene symbol. For the “Data Source: Combined” option, as all the datasets used in OSgbm already have been published, processed and normalized well, in order to avoid of the batch effect and platform biases among these datasets, we first stratify the patients into high- and low-expression group for the input gene in each dataset, and then merged relative patients from high- and low-expression group from each dataset into a combined high-expression group (Upper group in the Kaplan–Meier plot) and a combined low-expression group (Lower group in the Kaplan–Meier plot) for the analysis of Kaplan–Meier plot and log-rank test. The statistical analyses of input were performed with R package: KM curves with Hazard ratio (HR, 95% confidence interval) and log-rank *p* value were calculated by R package ‘survival’. OSgbm is available at http://bioinfo.henu.edu.cn/GBM/GBMList.jsp.

### Validation of Previously Reported Prognostic Biomarkers

A PubMed search was performed to identify previously reported GBM prognostic biomarkers, using keywords ‘glioblastoma’, ‘survival’ and ‘biomarker’. Totally, 53 prognostic biomarkers were identified from 2013 publications. The flow chart of biomarker collection was showed in **Figure S1**. The prognostic values of these published biomarkers were analyzed in either a form of combined cohorts of all GBM patients or in a single cohort in our database.

## Results

### The Clinical Characteristics of GBM Datasets Used in OSgbm

In OSgbm, we included a total of 684 unique GBM samples from seven datasets, including one TCGA cohort, four GEO cohorts and two CGGA cohorts. The survival information includes overall survival (OS), disease specific survival (DSS), disease free interval (DFI) and progression free interval (PFI) (Liu et al., 2018). The confounding clinical factors, such as age, grade, gender, histology and treatment regimens were included as well. Clinical characteristics of these datasets in the OSgbm were presented in **Table 1**. All of the 684 patients have OS data, and the median OS time was 13.44 months, while 153 GBM patients from TCGA cohort have four above mentioned survival terms (OS, DSS, DFI and PFI). The median age of all the patients is 50 years. The death rate is 78.49%. A large proportion of the patients are in grade IV, especially in the two CGGA datasets (99.28% and 100%, respectively).

**Table 1:** Clinical characteristics of each GBM dataset used in OSgbm.

Data Source	Sample Size (n)	Median Age (years)	Death (%)	OS Median (years)	Gender (male, %)	Grade (I/II/III/IV, %)	Survival Terms
TCGA	153	60	79.08	11.90	64.71	–	OS, DSS, DFI, PFI
GSE7696	80	52	81.25	15.58	73.75	–	OS
GSE4412	85	42	69.41	12.97	37.65	0/0/30.59/69.41	OS
GSE42669	57	51	80.70	14.93	52.63	–	OS
GSE30472	54	–	88.89	15.72	–	3.7/12.96/29.63/53.71	OS
CGGAseq	128	48	66.67	9.55	65.22	0/0/0.72/99.28	OS
CGGAarray	127	47	83.46	13.43	62.20	0/0/0/100	OS
Total	684	50	78.49	13.44	59.36	–	–

### Set-Up of OSgbm Web Server

The main function of OSgbm web server is to evaluate and determine the prognostic value of the quested genes. The users start by typing the gene symbol and choosing one dataset of interest or the combined dataset with pooling all the datasets together. To measure the association between a quested gene and survival, GBM samples are categorized according to the median (or other appropriate cutoff value, such as Trichotomy, Quartile) of the selected gene, and KM analysis is used to compare the outcomes between groups (Xie et al., 2019b). The user could limit the analysis in a subgroup of the patients by setting the age range, grade, gender and so on. Once the gene symbol is input and clinical characters are chosen, OS, DSS, DFI or PFI of each stratified group can be measured and analysis results will be available on the output web page. The prognostic value of each given gene is determined by HR (95% CI) and log-rank *p* value.

### Validation of Previously Reported GBM Prognostic Biomarkers

**Figure 1:**
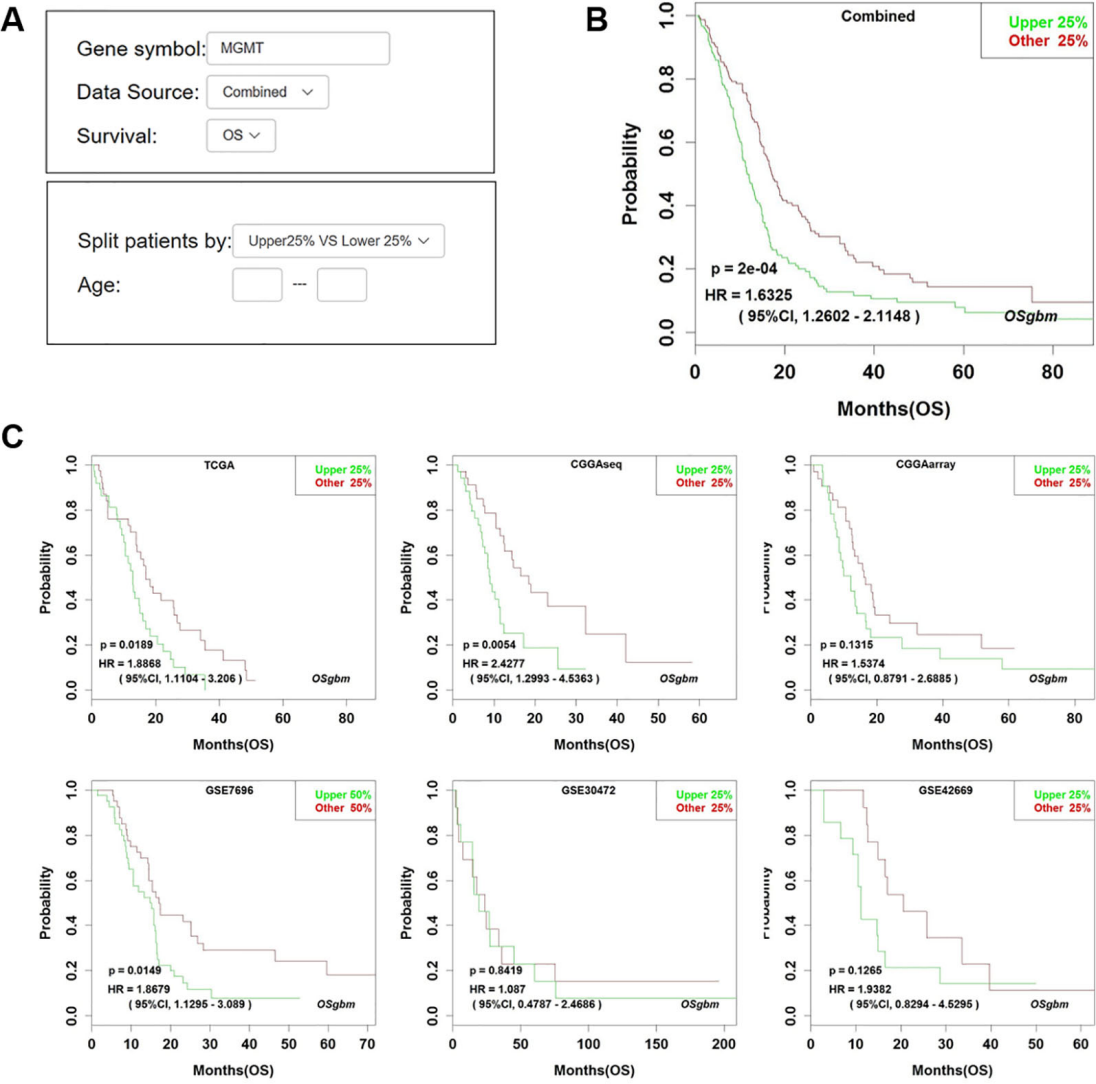
Analysis of the prognostic value of *MGMT* in OSgbm. **(A)** The options of input parameters used in the prognostic analysis of *MGMT* in OSgbm. **(B)** The output web page of prognosis analysis of *MGMT* using a combined cohort with pooling all datasets together in OSgbm. **(C)** The OSgbm output of gene *MGMT* in single cohort.

To determine the performance of this online tool, 53 previously published GBM prognostic factors collected as the procedure shown in **Figure S1** and then they were evaluated in OSgbm (**Table 2**, **Figure 1**) (Sano et al., 1999; Hung and Howng, 2003; Heimberger et al., 2005; Aaberg-Jessen et al., 2009; Shirai et al., 2009; Pu et al., 2011; Cui et al., 2013; De Tayrac et al., 2013; Lee et al., 2013; Rosati et al., 2013; Wang et al., 2013; Wu et al., 2013; Bai et al., 2014; Han et al., 2014; Meng et al., 2014; Olmez et al., 2014; Zupancic et al., 2014; Maris et al., 2015; Moutal et al., 2015; Yan et al., 2015; Zhao et al., 2015; Chaurasia et al., 2016; Nduom et al., 2016; Steponaitis et al., 2016; Steponaitis et al., 2016; Zhang et al., 2016a; Zhang et al., 2016b; Deng et al., 2017; Dong et al., 2017; Ge et al., 2017; Han et al., 2017; Haynes et al., 2017; Huang et al., 2017; Li et al., 2017; Luo and Zhuang, 2017; Ma et al., 2017; Mu et al., 2017; Roy et al., 2017; Wu et al., 2017; Zhang et al., 2017; Cai et al., 2018; Cetin et al., 2018; Cheng et al., 2018; Chou et al., 2018; Dahlrot et al., 2018; Gonçalves et al., 2018; Hayashi et al., 2018; Lv et al., 2018; Qian et al., 2018; Vasaikar et al., 2018; Hallal et al., 2019). OS was selected as the survival term. Among these prognostic genes, 51 of them showed significant prognostic ability in a large-scale combined cohort (33 genes) or in single cohort (18 genes), which were consistent with the prognostic value reported in the literature. The remaining two genes (*IGF1R* and *PCBP2*) display significant prognostic values in OSgbm, but is contradictory to what was reported in the literatures. Both of them were shown as favorable prognostic biomarkers in OSgbm but were reported to be unfavorable GBM prognostic biomarkers in previous reports (**Table 2**) (Maris et al., 2015; Luo and Zhuang, 2017).

**Table 2:** Validation of previously reported prognostic biomarkers in OSgbm.

Gene symbol	Validation results				Literature data				
	OS, HR (95% CI)	*p* Value	Cut Off	Osgbm	OS, HR (95% CI)	*p* Value	Sample (n)	Level	Reference
*PROM1*	2.412 (1.040–4.174)	0.007	Upper 25% vs Lower 25%	GSE7679	2.39 (1.77–3.23)	<0.001	656	mRNA	(Zhang et al., 2016)
*SRGN*	2.371 (1.256–4.477)	0.008	Upper 25% vs Lower 25%	CGGAseq	–	0.037	504	mRNA	(Roy et al., 2017)
*EDNRB*	2.272 (1.115–4.627)	0.024	Upper 25% vs Lower 75%	GSE30472	2.86 (1.12–7.34)	0.031	25	Protein	(Vasaikar et al., 2018)
*PSMB4*	2.074 (1.187–3.626)	0.010	Upper 25% vs Lower 25%	CGGAseq	–	<0.001	77	Protein	(Cheng et al., 2018)
*WNT6*	2.035 (1.098–3.770)	0.024	Upper 25% vs Lower 25%	CGGAseq	–	0.004	16	Protein	(Gonçalves et al., 2018)
*DPYSL5*	2.023 (1.160–3.527)	0.013	Upper 25% vs Lower 25%	CGGAarray	–	0.026	183	Protein	(Moutal et al., 2015)
*IL17A*	2.009 (1.107–3.646)	0.022	Upper 50% vs Lower 50%	GSE30472	–	0.007	41	Protein	(Cui et al., 2013)
*TLR9*	1.976 (1.089–3.588)	0.025	Upper 25% vs Lower 25%	CGGAseq	–	0.020	46	Protein	(Mu et al., 2017)
*ACKR3*	1.974 (1.040–3.747)	0.038	Upper 30% vs Lower 30%	GSE7679	1.56 (1.04–2.51)	0.03	146	Protein	(Deng et al., 2017)
*H19*	1.864 (1.309–2.653)	<0.001	Upper 25% vs Lower 25%	Combined	–	0.034	–	mRNA	(Wu et al., 2017)
*EGFR*	1.845 (1.077–3.160)	0.026	Upper 25% vs Lower 75%	GSE7696	–	<0.001	196	Protein	(Heimberger et al., 2005)
*NUSAP1*	1.748 (1.006–3.040	0.048	Upper 25% vs Lower 25%	CGGAarray	0.65 (0.49–0.86)*	0.003	518	mRNA	(Qian et al., 2018)
*CHAF1B*	1.707 (1.323–2.203)	<0.001	Upper 30% vs Lower 30%	Combined	–	0.004	96	Protein	(De Tayrac et al., 2013)
*TAGLN2*	1.665 (1.282–2.161)	<0.001	Upper 25% vs Lower 25%	Combined	–	<0.05	667	mRNA	(Han et al., 2017)
*BIRC1*	1.658 (1.266–2.172)	<0.001	Upper 25% vs Lower 25%	Combined	–	0.0003	66	Protein	(Shirai et al., 2009)
*MGMT*	1.633 (1.260–2.115)	<0.001	Upper 25% vs Lower 25%	Combined	1.50	0.01	157	Protein	(Dahlrot et al., 2018)
*CD70*	1.561 (1.180–2.065)	0.002	Upper 25% vs Lower 25%	Combined	1.6 (0.98–2.51)	0.046	107	mRNA	(Ge et al., 2017)
*CXCR4*	1.558 (1.207–2.010)	<0.001	Upper 25% vs Lower 25%	Combined	–	<0.05	156	mRNA	(Ma et al., 2017)
*CA9*	1.556 (1.202–2.015)	<0.001	Upper 25% vs Lower 75%	Combined	–	0.004	66	Protein	(Cetin et al., 2018)
*PDCD1*	1.508 (1.171–1.942)	0.002	Upper 30% vs Lower 30%	Combined	–	0.028	149	mRNA	(Nduom et al., 2016)
*IDH1*	1.490 (1.013–2.192)	0.043	Upper 50% vs Lower 50%	CGGAarray	–	0.045	163	Protein	(Chaurasia et al., 2016)
*IGFBP2*	1.467 (1.132–1.902)	0.004	Upper 25% vs Lower 25%	Combined	1.04 (1.02–1.05)	0.001	83	Plasma	(Han et al., 2014)
*PBK*	1.456 (1.131–1.875)	0.004	Upper 25% vs Lower 25%	Combined	–	0.007	32	Protein	(Hayashi et al., 2018)
*EFEMP2*	1.446 (1.117–1.871)	0.005	Upper 25% vs Lower 25%	Combined	–	<0.01	77	mRNA	(Li et al., 2017)
*MET*	1.434 (1.130–1.820)	0.003	Upper 30% vs Lower 30%	Combined	1.7 (1.1–2.2)	<0.05	69	Protein	(Olmez et al., 2014)
*CHI3L1*	1.438 (1.104–1.872)	0.007	Upper 25% vs Lower 25%	GSE30472	–	<0.01	98	mRNA	(Steponaitis et al., 2016)
*TRAF2*	1.443 (1.118–1.863)	0.005	Upper 25% vs Lower 25%	Combined	–	0.03	105	mRNA	(Zhang et al., 2017)
*HMGB2*	1.391 (1.099–1.759)	0.006	Upper 30% vs Lower 30%	Combined	3.35 (1.25–9.02)	0.017	51	Protein	(Wu et al., 2013)
*MCM6*	1.387 (1.132–1.699)	0.002	Upper 25% vs Lower 75%	Combined	1.19	0.006	325	mRNA	(Cai et al., 2018)
*CD44*	1.386 (1.073–1.790)	0.012	Upper 25% vs Lower 25%	Combined	–	<0.001	28	Protein	(Steponaitis et al., 2016)
*TIMP1*	1.342 (1.025–1.758)	0.033	Upper 25% vs Lower 25%	Combined	3.2 (1.5–6.7)	0.004	112	Protein	(Aaberg-Jessen et al., 2009)
*CD151*	1.336 (1.023–1.746)	0.034	Upper 25% vs Lower 25%	Combined	5.064 (1.427–17.969)	0.012	211	Protein	(Lee et al., 2013)
*TWIST1*	1.312 (1.013–1.699)	0.039	Upper 25% vs Lower 25%	Combined	5.745 (1.331–1.89)	0.017	86	Protein	(Wang et al., 2013)
*CCT6A*	1.316 (1.045–1.655)	0.019	Upper 30% vs Lower 30%	Combined	3.21 (2.85–3.65)	0.006	497	Protein	(Hallal et al., 2019)
*APC*	1.308 (1.093–1.566)	0.004	Upper 50% vs Lower 50%	Combined	–	<0.001	83	Protein	(Rosati et al., 2013)
*CD247*	1.292 (1.022–1.633)	0.032	Upper 30% vs Lower 30%	Combined	1.54 (1.05–2.28)	0.023	149	mRNA	(Nduom et al., 2016)
*CXCR3*	1.272 (1.027–1.575)	0.028	Upper 25% vs Lower 75%	Combined	1.56 (1.04–2.51)	0.03	146	Protein	(Pu et al., 2011)
*TCTN1*	1.223 (1.011–1.493)	0.039	Upper 30% vs Lower 70%	Combined	1.32 (1.08–1.61)	0.006	518	mRNA	(Meng et al., 2014)
*BICD1*	0.794 (0.644–0.978)^#^	0.030	Lower 25% vs Upper 75%	Combined	1.577 (1.299–1.914)	<0.001	523	mRNA	(Huang et al., 2017)
*IFIT1*	0.770 (0.609–0.973)	0.029	Upper 30% vs Lower 30%	Combined	0.22 (0.10–0.52)	0.001	70	mRNA	(Zhang et al., 2016)
*BRMS1L*	0.753 (0.587–0.966)	0.026	Upper 25% vs Lower 75%	Combined	–	<0.05	60	mRNA	(Lv et al., 2018)
*IGF1R*	0.745 (0.588–0.944)	0.015	Upper 30% vs Lower 30%	Combined	1.65 (1.10–2.47)	0.016	167	Protein	(Maris et al., 2015)
*GANO1*	0.748 (0.585–0.957)	0.021	Upper 30% vs Lower 30%	Combined	–	0.009	178	Protein	(Zupancic et al., 2014)
*PTEN*	0.729 (0.567–0.938)	0.014	Upper 25% vs Lower 25%	Combined	3.3 (1.6–4.3)*	0.0003	61	mRNA	(Sano et al., 1999)
*SEMA6A*	0.694 (0.556–0.867)	0.001	Upper 25% vs Lower 75%	Combined	1.71 (1.01–2.65)*	0.012	200	Protein	(Zhao et al., 2015)
*PHF3*	0.683 (0.529–0.883)	0.004	Upper 25% vs Lower 25%	Combined	0.44 (0.26–0.77)	0.0031	35	Protein	(Yan et al., 2015)
*PPARα*	0.644 (0.503–0.825)	<0.001	Upper 30% vs Lower 30%	Combined	1.31 (1.05–1.63)*	0.016	473	mRNA	(Haynes et al., 2017)
*PCBP2*	0.632 (0.417–0.957)	0.031	Upper 25% vs Lower 75%	TCGA	–	<0.001	130	mRNA	(Luo and Zhuang, 2017)
*LAPTM4B*	0.626 (0.433–0.894)	0.010	Upper 50% vs Lower 50%	TCGA	–	<0.001	39	Protein	(Dong et al., 2017)
*ANXA7*	0.619 (0.475–0.806)	<0.001	Upper 25% vs Lower 25%	Combined	–	<0.001	99	Protein	(Hung and Howng, 2003)
*PHF20*	0.557 (0.319–0.972)	0.040	Upper 50% vs Lower 50%	CGGAarray	0.5 (0.29–0.86)	0.012	62	Protein	(Yan et al., 2015)
*TES*	0.407 (0.173–0.958)	0.040	Upper 30% vs Lower 30%	GSE42669	–	<0.05	37	Protein	(Bai et al., 2014)
*LGALS1*	0.368 (0.157–0.863)	0.022	Upper 25% vs Lower 25%	GSE42669	–	0.009	45	Protein	(Chou et al., 2018)

*: The lower gene expression compared with higher gene expression in the literature data.

^#^: The lower gene expression compared with higher gene expression in the OSgbm data.

## Discussion

The development of prognostic biomarkers is important for guiding the treatments especially for therapy-resistant GBM patients. In our work, we developed a new web server, OSgbm, to help researchers to evaluate the prognostic value of a given gene for GBM patients. OSgbm is easy to use and requires no special skills (such as bioinformatics training). With filtering by one or several clinical confounding factors provided in OSgbm, users can also evaluate the prognostic value of their interested genes according to their special needs. The function and performance tests of OSgbm web server showed that 96% (51 out of 53) of previously reported prognostic biomarkers could be confirmed in OSgbm, which indicates that these biomarkers validated in independent cohorts have the potency of translating to clinical applications, and also indicates the well performance of OSgbm. Nevertheless, there are two genes including *IGF1R* and *PCBP2* which showed different prognostic values to the literatures, the discrepancy of prognostic performance of *IGF1R* and *PCBP2* between OSgbm and literatures may be caused by race, different cohort size, or analysis level and methods (mRNA vs. protein, gene microarray vs. immunohistochemistry) (Maris et al., 2015; Luo and Zhuang, 2017). For example, the race reported in literatures for *PCBP2* is Asian, while that in validated cohort of OSgbm is mostly White. The mRNA level was analyzed in OSgbm for *IGF1R*, while IGF1R was determined by immunohistochemistry in literature. In addition, the race analyzed in OSgbm for *IGF1R* is Asian (Korea for GSE42669 and Chinese for CGGA), while the race reported in literature for IGF1R is European. As a result, it will be necessary to validate the prognostic performance of *IGF1R* and *PCBP2* in a larger independent cohort of glioblastoma.

In conclusion, OSgbm is a user-friendly web server to help researchers and clinicians to identify suitable prognostic biomarkers in GBM. Furthermore, we will keep update the database of OSgbm to collect more and more GBM datasets when new GBM dataset is available, and will implement the multivariate cox proportional hazards model into OSgbm for the purpose of adjustment for the confounding clinical factors, and we also encourage users to contact us to upload their own data into OSgbm.

## Data Availability Statement

All datasets for this study are included in the article/**Supplementary Material**.

## Author Contributions

XG conceived and directed the project. HD and QW collected data and developed the web server. HD, NL, JL, LG, MY, GZ, YA, FW, LX, and YL performed data analysis. WZ, HZ, and MZ contributed to data analysis and paper writing. XG and HD wrote the manuscript with the assistance and approval of all authors.

## Conflict of Interest

Author MZ is employed by company of Nanjing Jiliang Biotechnology Co., Ltd.

The remaining authors declare that the research was conducted in the absence of any commercial or financial relationships that could be construed as a potential conflict of interest.
