# Supplementary figures and images for: OSgbm: An Online Consensus Survival Analysis Web Server for Glioblastoma
Source: Front Genet. 2020 Feb 21;10:1378. doi: 10.3389/fgene.2019.01378 (PMC7046682; doi:10.3389/fgene.2019.01378)

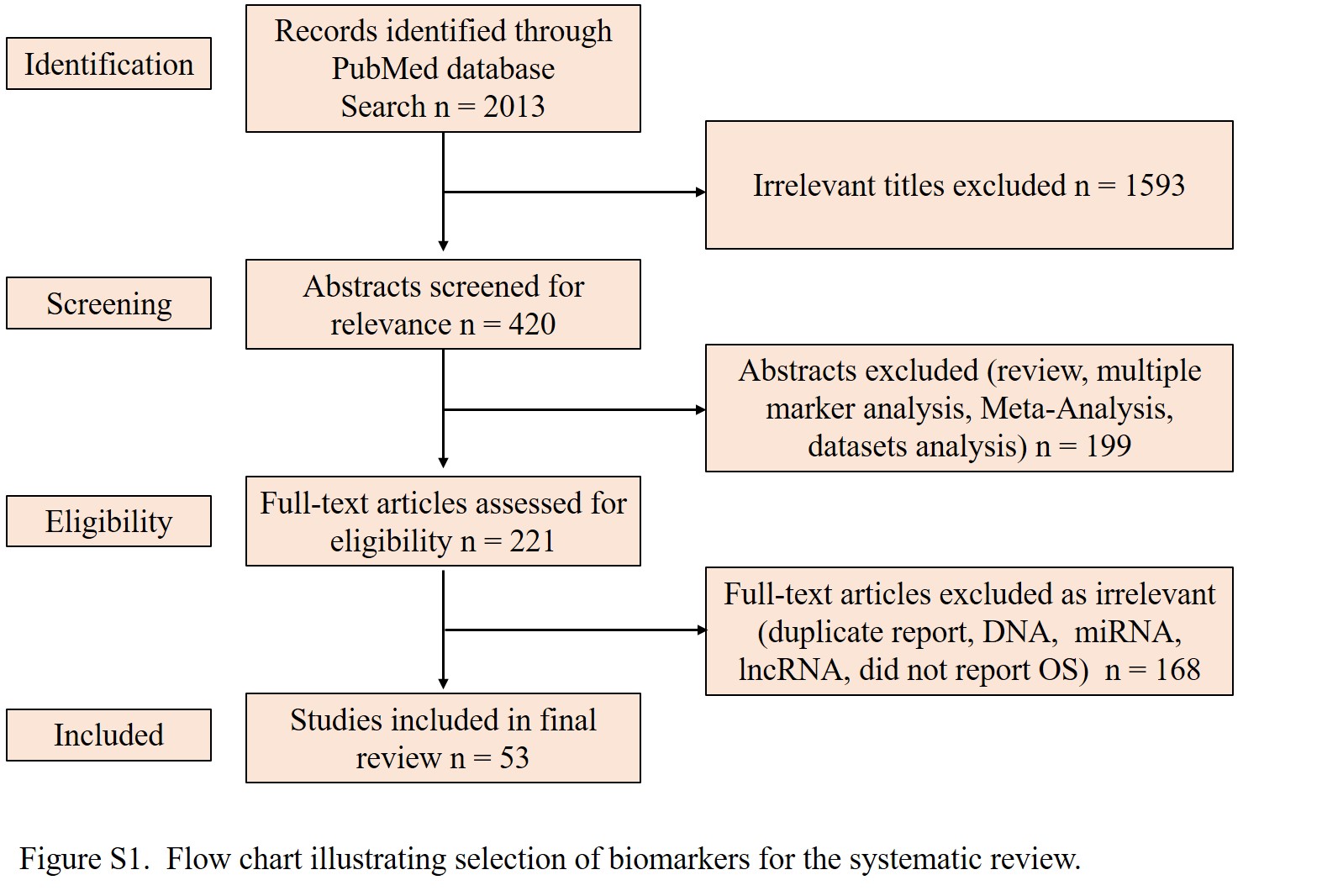

Supplement: Supplementary file 1 [file Image_1.jpeg]
